# A systematic review of eye-tracking technology in electrocardiogram interpretation research

**DOI:** 10.1186/s40001-025-03635-8

**Published:** 2025-12-05

**Authors:** Reza Amani-Beni, Bahar Darouei, Nasim Kakavand, Reza Eshraghi, Arsham Seifnezhad, Mohammad Reza Movahed, Athar Omid

**Affiliations:** 1 Medical Education Research Center, Department of Medical Education, Isfahan University of Medical Sciences, Isfahan, Iran; 2 Isfahan Cardiovascular Research Center, Cardiovascular Research Institute, Isfahan University of Medical Sciences, Isfahan, Iran; 3 Anesthesiology and Critical Care Research Center, Isfahan University of Medical Sciences, Isfahan, Iran; 4 School of Medicine, Isfahan University of Medical Sciences, Isfahan, Iran; 5 Social Determinants of Health Research Center, Isfahan University of Medical Sciences, Isfahan, Iran; 6 Department of Medicine, University of Arizona College of Medicine, Phoenix, USA; 7 Department of Medicine, University of Arizona Sarver Heart Center, Tucson, AZ USA

**Keywords:** Eye-tracking technology, Electrocardiography, Education, Medical, Clinical competence, Eye movements

## Abstract

**Background:**

Eye-tracking technology provides an objective method for analyzing visual behavior during diagnostic tasks such as electrocardiogram (ECG) interpretation. As a high-stakes and visually complex clinical skill, ECG interpretation benefits from technologies that differentiate experts from novices via gaze patterns. We systematically reviewed eye-tracking in ECG research to examine links between gaze metrics and diagnostic performance and to appraise educational applications.

**Methods:**

Following PRISMA (PROSPERO CRD420251052940), we searched PubMed, Embase, Web of Science, Scopus, ERIC, IEEE Xplore, and PubPsych databases to September 2025. Eligible studies used eye-tracking during ECG interpretation and reported visual behavior or diagnostic outcomes. Two independent reviewers performed the study selection, data extraction, and quality assessment using the Jadad and Newcastle–Ottawa scales. Given heterogeneity, we conducted a narrative synthesis.

**Results:**

Nineteen studies (573 participants) were included, mainly involving students or early-career clinicians. Common metrics included fixation duration, counts, time to first fixation, and scan-path efficiency. Experts showed faster, more targeted fixation on critical leads (V1, V2, and II), shorter interpretation times, and higher accuracy. Novices exhibited scattered visual behavior and delayed attention to diagnostically relevant regions. Eye movement data were informative of underlying cognitive processes, including the selection, organization, and integration of ECG features. Additional factors, such as presentation format, individual experience, metacognitive strategies, and emotional responses, may moderate gaze behavior, although these were underexplored. Educational interventions leveraging expert gaze modeling or structured checklists showed mixed but promising outcomes.

**Conclusions:**

Eye-tracking differentiates expertise in ECG interpretation and may inform diagnostic reasoning, but prospective validation is needed. Gaze metrics are candidate indicators of performance and a foundation for feedback-based educational tools. Standardized protocols, larger multi-site studies, and inclusion of diverse learners are required to realize the potential of eye-tracking in ECG training and assessment.

**Graphical Abstract:**

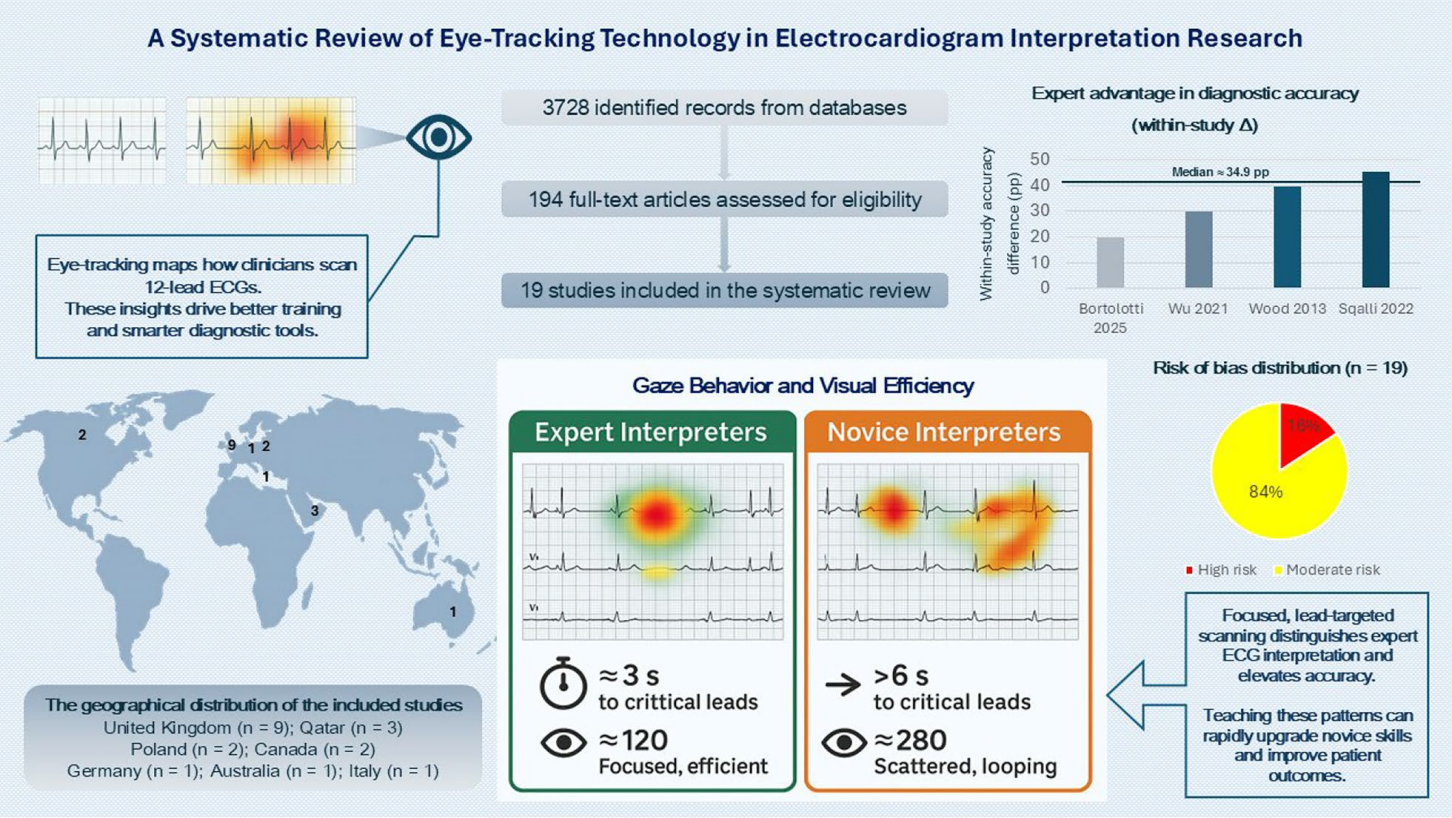

**Supplementary Information:**

The online version contains supplementary material available at 10.1186/s40001-025-03635-8.

## Background

Eye-tracking technology has emerged as a powerful method for understanding how individuals process visual information, particularly in domains requiring complex perceptual-cognitive integration, such as clinical decision-making [1]. Eye-tracking technology originated in the nineteenth century and initially served as a tool to study how individuals process written texts during reading [2]. In medical education, eye-tracking has gained traction due to its ability to objectively differentiate expert and novice performances based on gaze behavior during diagnostic tasks.

Electrocardiogram (ECG) interpretation is a fundamental skill in medical practice that requires pattern recognition, spatial reasoning, and selective attention across multiple leads [3, 4]. The electrical activity of the heart is recorded by placing electrodes on the skin surface [5]. The 12-lead ECG provides complementary views of cardiac activation: limb leads (I, II, III, aVR, aVL, aVF) record activity in the vertical plane, while precordial leads (V1-V6) record the horizontal plane [6]. Accordingly, V1-V4 emphasize anterior/septal regions, II/III/aVF the inferior wall, and V5-V6 the lateral territory; so, attention to specific leads maps to anatomic territories [7].

ECG is a vital diagnostic instrument in emergency medicine that offers critical information about a patient’s cardiac condition [8]. However, ECG interpretation remains a challenging competency, with obstacles to achieving fluency arising at various stages of medical education [3]. Studies have consistently revealed substantial variability in ECG interpretation accuracy across experience levels, with medical students averaging only 42% accuracy compared to 75% among cardiologists [9]. Combined with the considerable variation in how ECG interpretation is taught across institutions [10], this highlights the need for stronger evidence to inform effective educational practice. In addition, as ECGs have become more affordable and accessible, they are increasingly being used as routine diagnostic tools in clinical practice [11].

Common methods for teaching ECG often rely on structured checklists that can burden novice learners [12, 13]. Interestingly, as expertise increases, clinicians tend to abandon rigid schemas in favor of more efficient and intuitive strategies [4, 14]. Eye-tracking offers a promising alternative by visualizing expert gaze patterns, enabling learners to observe and internalize expert diagnostic behavior directly [13].

Despite growing interest, no prior systematic review has synthesized the current body of evidence on eye-tracking applications in ECG interpretation. This review aims to fill this gap by systematically evaluating studies that have employed eye-tracking to analyze visual behavior during ECG reading tasks. In this study, we aimed to highlight the diagnostic implications of gaze behavior, the educational utility of eye-tracking feedback, and future research directions in visual expertise assessment. Because expert advantages in ECG reading manifest as quantifiable gaze behaviors, eye-tracking provides machine-readable features that enable objective assessment, adaptive tutoring interfaces, and predictive modeling of expertise, which can bridge the gap between medical education and engineering.

## Methods

### Registration and reporting guidelines

This systematic review was conducted by the Preferred Reporting Items for Systematic Reviews and Meta-Analyses (PRISMA) guidelines [15]. The study protocol was prospectively submitted and registered in PROSPERO with the registration identifier [CRD420251052940].

### Search strategy

A comprehensive literature search was conducted in five core biomedical databases (PubMed, Embase, Web of Science, Scopus, and ERIC) and supplemented by IEEE Xplore and PubPsych to capture engineering and psychology/human-factors studies. Searches covered inception to December 24, 2024, and were updated through September 20, 2025. The search was designed to identify studies that used eye-tracking technology for ECG interpretation. Key terms included combinations of “eye-tracking”, “gaze behavior”, “electrocardiogram”, “ECG interpretation”, and “visual attention”. In addition to database searching, we conducted supplementary searches using Google Scholar and performed a manual search of the reference lists from the included articles to identify any additional eligible studies. The full search strategy is presented in Table S1.

### Study selection

All the identified records were imported into EndNote for deduplication, and the remaining entries were screened using a two-stage process. In the first phase, two reviewers independently screened the titles and abstracts of the retrieved studies. In the second phase, the full texts of potentially eligible studies were reviewed against predefined inclusion criteria. Disagreements were resolved through discussion or adjudication by a third reviewer if necessary.

### Eligibility criteria

Studies were eligible for inclusion if they involved human participants, including laypersons, medical students, and healthcare professionals, who were engaged in ECG interpretation. Eligible studies were required to employ eye-tracking technology during the ECG interpretation process and to report at least one of the following outcomes: eye movement metrics (such as fixation duration or scan-path characteristics), interpretation time, or diagnostic accuracy. The acceptable study designs included experimental, quasi-experimental, and observational studies that presented the original data. Only full-text articles published in peer-reviewed journals were included in this study. We applied no language restrictions. Studies were excluded if they were reviews, editorials, abstracts without full text, non-ECG-related eye-tracking studies, animal studies, or articles in which the full text was not available.

### Data extraction

Data were extracted using a pre-structured Microsoft Excel spreadsheet. We emphasized engineering details needed for reproducibility, alongside participant and study design information. Specifically, we captured the first author, year of publication, country, study design, sample size, participant groups by ECG expertise, age of participants, eye-tracking device type, the number and type of ECGs used, diagnostic categories, the specific ECG task, fixation-related metrics, accuracy data, analysis methods, and key findings. Data extraction was independently conducted by two reviewers, and discrepancies were reconciled through discussion with a third reviewer.

### Quality assessment

The methodological quality of the included studies was evaluated using two established tools: The Jadad scale for randomized controlled trials [16] and the Newcastle–Ottawa Scale (NOS) for cohort and observational studies [17]. The Jadad scale assesses studies based on criteria such as randomization, blinding, and clarity of outcome reporting, with scores ranging from 1 to 5. The NOS rates study quality on a 0–9 scale, considering factors such as participant selection, group comparability, and the validity of outcome assessment. To facilitate comparisons across study designs, quality ratings were categorized as follows: poor (Jadad 1–2; NOS 0–5), moderate (Jadad 3; NOS 6–7), or good (Jadad 4–5; NOS 8–9) [18].

### Data synthesis

Given the substantial heterogeneity in study design, outcome metrics, ECG tasks, and eye-tracking technology used, a meta-analysis was not performed. The results were qualitatively analyzed. The studies were grouped thematically according to the outcomes they reported, including visual search patterns (e.g., fixations and scan paths), interpretation time, diagnostic accuracy, and the relationship between gaze behavior and diagnostic performance. Within each theme, the results were summarized and compared across studies.

In addition to narrative synthesis, we computed within-study differences in diagnostic accuracy between experts and the least-experienced comparator group (Δ = expert proportion − comparator proportion). When accuracy was reported as scores out of a fixed total, scores were normalized to proportions. We summarized the direction of effect across studies and reported the range (and median) of within-study differences. As a sensitivity check, we repeated the summary excluding the largest and smallest Δ.

## Results

### Overview of study selection and included studies

**Fig. 1:**
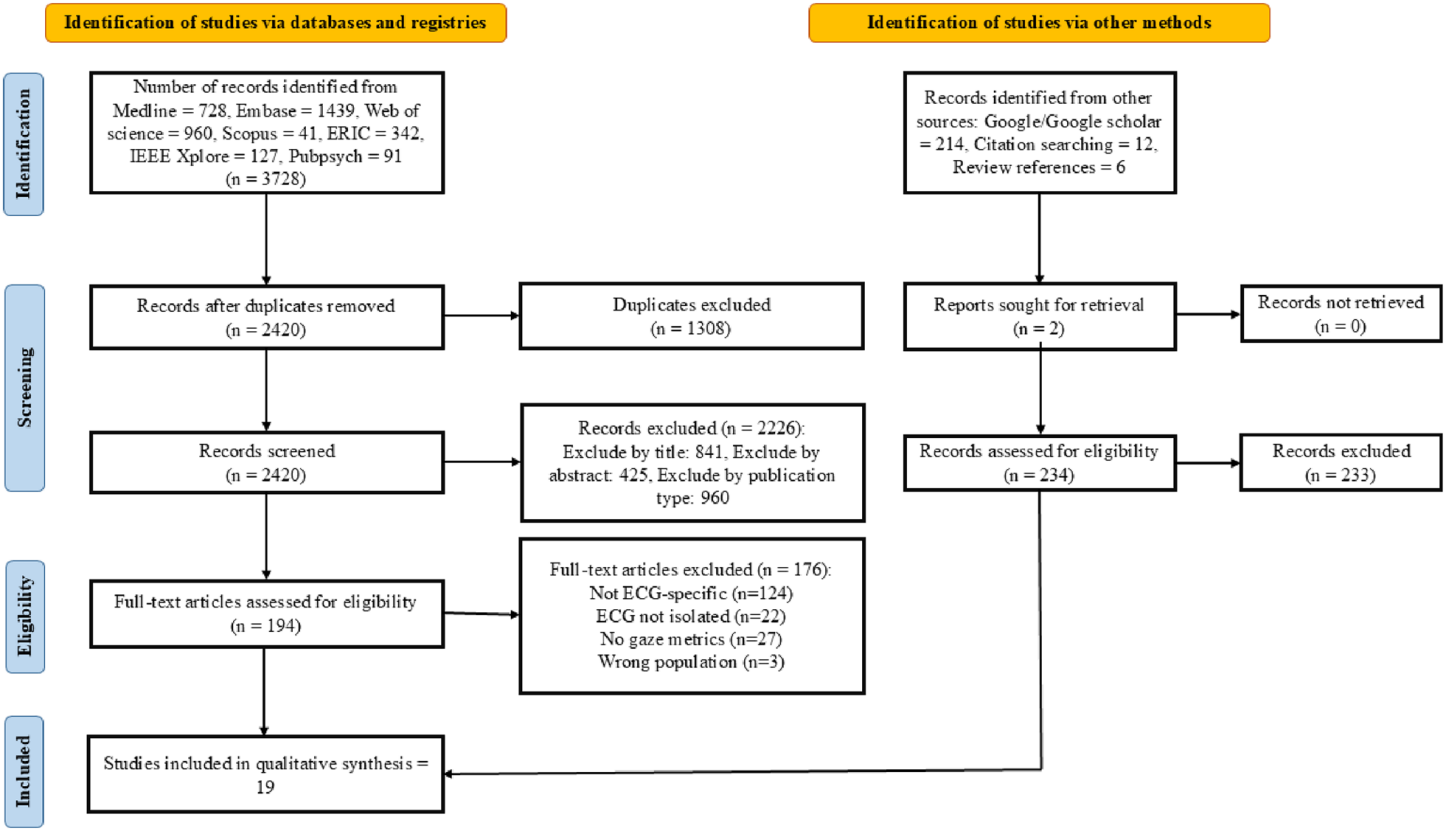
PRISMA flow diagram of study selection

Database searches identified 3,728 records (Medline: 728, Embase: 1,439, Web of Science: 960; Scopus: 41, ERIC: 342, IEEE Xplore: 127; PubPsych: 91). After removing duplicates, 2,420 records were screened. After screening by title/abstract, 194 studies were assessed by full text. Of these, 176 were excluded for various reasons, leading to the inclusion of 18 studies. In addition, 232 records were identified via other methods (Google/Google Scholar, citation searching, reference lists); 234 full texts were assessed from these sources, yielding one additional eligible study. In total, 19 studies met the inclusion criteria and were included in the qualitative synthesis [4, 13, 19–36]. The PRISMA flow diagram illustrates the study selection process (Fig. 1). The geographical distribution of the studies was notably concentrated in the United Kingdom (n = 9) [4, 19, 23–26, 28–30], which accounted for half of the total studies, followed by Qatar (n = 3) [32–34], Poland (n = 2) [20–22], Canada (n = 2) [31, 35], Germany (n = 1) [13], Australia (n = 1) [27], and Italy (n = 1) [36] (Table 1). Methodologically, the studies included experimental trials, observational designs, exploratory investigations, and randomized clinical trials. The sample sizes varied widely, from single expert case analyses [23, 25] to studies involving 91 participants [13]. Of the 19 included studies, three were rated as having a high risk of bias [23, 30, 35]. The remaining 16 studies were assessed as having a moderate risk (Table 1).

**Table 1:** Summary of the baseline characteristics of the included studies

First author, year	Country	Study type	Sample size	Study groups based on ECG Expertise	Mean age	ECG diagnoses, (n)	Task	Analysis	ROB
Alahmadi et al. (2019) [19]	UK	Experimental trial	30 (15 men, 15 women)	Laypeople (no ECG experience)	26	QT prolongation (7)	Detect QT differences across 3 presentation formats (21 trials)	Just noticeable difference (JND) psychometrics; fixation metrics (% fixated, duration); AOI heatmaps	Moderate
Augustyniak et al. (2003, 2006) [20, 22]	Poland	Experimental trial	38 (17 experts, 21 students)	Experts (12 ± 4 years experience) vs students (basic knowledge)	NA	Not specified (8 traces/observer)	ECG interpretation with eye-tracking	Scanpath stats (idle, total time, foveation %, attention density); trajectory/attention maps	Moderate
Augustyniak et al. (2005) [21]	Poland	Experimental	38 (17 experts, 21 students)	Experts (12 ± 4 years) vs students (basic knowledge)	NA	Not specified (8 traces/observer)	ECG interpretation with eye-tracking	Fixation detection from raw scanpaths; foveation-to-cardiac-event mapping	Moderate
Bond et al. (2012) [25]	UK	Observational study	1 clinical scientist	ECG expert	NA	MI (10), LVH (10), LBBB (9)	12-lead ECGs (3 × 4 layout); think-aloud + eye-tracking	Heatmaps; time per lead	Moderate
Bond et al. (2014) [26]	UK	Experimental observational study	21 expert annotators (19 men, 2 women)	Experts (31 ± 17 years experience; mode > 200 ECGs in last year)	61	STEMI (3), hypertrophy (3), arrhythmias (3), misplacement/dextrocardia (3)	Interpret 12 ECGs (balanced difficulty); think-aloud	Heatmaps; fixation duration; time-to-first-fixation; accuracy vs experience	Moderate
Bond et al. (2015) [23]	UK	Observational study	1 (Dr. Rory Childers)	Eminent expert cardiologist	NA	Not specified (12)	ECG interpretation + think-aloud	Heatmaps; fixation duration	High
Bortolotti et al. (2025) [36]	Italy	Observational pilot, cross-sectional	8 (4 tiers: PGY1–3, consultants)	Residents PGY1-3; board-certified cardiologists	32.5	STEMI, NSTEMI-OMI, Noncardiac chest pain, HF exacerbation (9)	12-lead interpretation (suspected ACS)	Between-group comparisons; fixation/pupil metrics; accuracy; heatmaps	Moderate
Breen et al. (2014) [26]	UK	Randomized controlled trial	33 (5 men, 28 women)	Eye-tracking feedback (n = 16) vs traditional teaching (n = 17)	24.5	10 (NSR, SB, ST, RAE, RVH, LVH + 1° AVB, WPW, nodal/brady, LAE + VE, VT, VF)	Interpret 10 ECGs; feedback by arm	Heatmaps/gaze paths; Mann–Whitney; Pearson r	Moderate
Broadbent et al. (2013) [27]	Australia	Exploratory study	49 nurses (22 novice nurses, 18 final-year students, 9 expert nurses)	Novices; final-year; experts	NA	AV block, NSR, anterior MI, inferior MI (4)	12-lead interpretation + multiple-choice questions	Heatmaps/gaze plots/AOIs; time-to-first-fixation; fixation count; visit duration	Moderate
Davies et al. (2016) [28]	UK	Exploratory study	31 (23 women, 8 men)	Average experience: 9.3 years, median: 6 years: 20 Cardiac physiologist/ technician, 4 Student cardiac physiologist /technician, 2 Cardiac registrar 1 for each following groups: Registrar, Medical student, Senior nurse, Consultant, FY2 doctor, and Health care assistant	NA	Layout type A: Anterolateral STEMI, atrial flutter, hyperkalemia, TdP, WPW Layout type B: LBBB, NSR, ST, SVT, ventricular paced rhythm, VT (11)	Interpret 11 ECGs (two layouts)	Markov transitions; fixation duration/count; Jensen–Shannon distance	Moderate
Davies et al. (2018) [29]	UK	Exploratory study	31 (23 women, 8 men)	19 Cardiac physiologists/technicians; 7 doctors/nurses; 5 Students	NA	Atrial flutter, anterolateral STEMI, hyperkalemia, LBBB, NSR, ST, SVT, ventricular paced rhythm, TdP, VT, WPW (10)	Interpret 11 ECGs; correctness by 2 experts	Scanpath length; Levenshtein distance; transition frequency	Moderate
Davies et al. (2019) [30]	UK	Exploratory study	31 (18 women, 13 men)	16 Physiologists/technicians; 2 Doctors; 2 nurses; 7 students 4 others	28	Anterolateral STEMI, LBBB, lateral STEMI, AF, RBBB, inferior STEMI with AF, anterior STEMI, high lateral STEMI, and inferolateral STEMI (9)	Interpret with/without clinical history	Grid-level transitions (lead/waveform); fixation metrics	High
Scherff et al. (2024) [13]	Germany	Randomized controlled trial	91 medical students (76% women)	Intervention (EYE-ECG, n = 47): 9-min video of expert real-time ECG interpretation with eye-tracking overlay and synchronized CRR commentary (15clips) Control (TAU, n = 41): standard case-based ECG training	24.1	Not specified (13—4 clinical cases and 9 post-test ECG scenarios)	~ 5-h online training; pre/post tests	Multivariable regression; engagement ratings	Moderate
Sibbald et al. (2015) [31]	Canada	Experimental	16 cardiology residents (PGY 4–6 years of post-graduate experience)	Checklist vs analytic-prompt verification (CP vs. AP)	NA	Acute infarction, VT, etc. (12)	Interpret 12 ECGs; verify with checklist vs prompt	Eye-tracking during verification; pupil/saccades/dwell; error-correction rates	Moderate
Sqalli et al. (2022) [33]	Qatar	Experimental study	63 participants (51 men,12 women)	9 Junior medical students; 10 senior medical students; 14 nurses, technicians 11 fellows; 9 cardiology consultants (1–15 yrs of ECG experience)	28	AF, atrial flutter, ventricular paced rhythm, ST-elevation, NSR, LBBB, VT, hyperkalemia, CHB, and WPW (10)	Interpret 10 ECGs (30 s each)	Fixation count/revisits; accuracy	Moderate
Sqalli et al. (2023) [32]	Qatar	Experimental study	63 participants (medical students and practitioners)	Junior medical students, senior medical students, nurses, technicians, fellows, and cardiology consultants	28	Not specified (630)	Interpreted 10 different ECGs, 30 s each; ML expertise prediction	Correlations + ML models (SVM, etc.)	Moderate
Sqalli et al. (2021) [34]	Qatar	Experimental study	16 medical students (15 males, 1 female)	10 Clinical (senior) students vs. 6 Preclinical (junior) students	21—24	NSR, AF, hyperkalemia, atrial flutter, VT, WPW, ventricular paced rhythm, LBBB, STEMI, CHB (160)	Interpreted 10 different ECGs, 30 s each	Heatmaps; fixation duration; revisitation	Moderate
Wood et al. (2013) [4]	UK	Experimental study	20 participants (10 final-year medical students, 10 consultant emergency physicians)	Novices vs experts	NA	Inferior STEMI, 1° AVB, AF, long-QT, Inferior/ inferolateral MI, Anterior/ anteroseptal MI, NSR (16)	Interpret 16 ECGs (13 abnormal, 3 normal) Half of the ECGs included brief clinical histories	1) Eye-tracking heatmaps 2) Dwell time on critical leads vs. overall ECG scanning	Moderate
Wu et al. (2021) [35]	Canada	Mixed-methods of quantitative eye-tracking study with retrospective interviews	30 (,21 men, 9 women)	10 Medical students (3 years since medical school); 10 EM residents (PGY 1–4, 5.3 years) 10 EM attendings (17.9 years)	NA	NSR, hypothermia, hyperkalemia, and benign early repolarization (10)	Interpret 10 ECGs (online library); re-situ interviews	Time-to-diagnosis; accuracy; heatmaps; thematic reasoning analysis	High

*ACS* Acute Coronary Syndrome, *AV block* Atrioventricular Block, *AF* Atrial Fibrillation, *AOI* Area of Interest, *CHB* Complete Heart Block, *CH* Checklist, *CRR* Clinical Reasoning Report, *CR* Correct Rejection, *ECG* Electrocardiogram, *EM* Emergency Medicine, *FA* False Alarm, *FY2* Foundation Year 2, *LBBB* Left Bundle Branch Block, *LVH* Left Ventricular Hypertrophy, *MI* Myocardial Infarction, *ML* Machine Learning, *NA* Not Available, *NSR* Normal Sinus Rhythm, *NSTEMI-OMI* non ST-elevation myocardial infarction with occlusion, *RBBB* Right Bundle Branch Block, *ROB* Risk of Bias, *STEMI* ST-elevation myocardial infarction, *ST* Sinus Tachycardia, *SVT* Supraventricular Tachycardia, *TAU* Treatment As Usual, *VF* Ventricular Fibrillation, *VT* Ventricular Tachycardia, *WPW* Wolff-Parkinson-White Syndrome

Participant demographics were notably heterogeneous, comprising over 573 individuals with a broad spectrum of ECG interpretation expertise. These ranged from complete novices, such as laypersons and preclinical medical students, to highly experienced clinicians, including cardiology consultants, cardiac physiologists, emergency physicians, and expert annotators (Table 1). For example, Alahmadi et al. involved 30 laypersons [19], whereas Bond et al. included 21 expert annotators [24]. In contrast, Sqalli et al. conducted one of the largest multilevel comparisons, enrolling 63 participants distributed across five distinct expertise levels: junior and senior medical students, nurses, technicians, fellows, and cardiology consultants [32]. Eight studies reported a mean age of 24–35 years, particularly those involving students and early career clinicians. Bond et al., involving senior experts, reported a higher average age of 61± 17 years among expert annotators [24] (Table 1).

Engineering reporting was uneven across studies. The device model used was commonly stated, whereas sampling rate, calibration, display parameters, synchronization, lead-area templates, blink handling, and data-loss were rarely reported. The most frequently used system was the Tobii series, featured in 13 studies [19, 23–30, 32–35]. The Tobii models included Tobii X60, X120, X2-60, Pro X2-60, and Tobii 1750. Higher-resolution systems, such as the Tobii Pro X3-120 (sampling rate: 120 Hz), were used in later studies [35, 36]. Two studies employed the OBER-2 infrared eye-tracker, a custom-built device used in early visual scanning research [20, 21]. One study utilized the ASL Mobile Eye system [4], whereas another employed SensoMotoric Instruments BeGaze 2.4 [31].

The number of ECGs interpreted in each study varied significantly. Some investigations have employed as few as four to seven ECGs for focused diagnostic tasks [19, 27], while Sqalli et al. analyzed up to 630 ECGs in large-scale experiments using machine-learning approaches [32]. Most studies were within the 10–30 ECGs range (11 studies). These ECGs covered a broad spectrum of conditions, including normal sinus rhythm, atrial fibrillation, ST-elevation myocardial infarction (STEMI), left bundle branch block, ventricular tachycardia, and QT interval abnormalities (Table 1).

Technical reporting varied substantially across studies, with sampling rates ranging from 60 to 1000 Hz, and calibration accuracy and detector details often being unreported (Table S2).

### Gaze behavior and visual efficiency among experts

Expert interpreters followed a rapid and highly focused visual strategy. They oriented to diagnostically critical leads, most often V1, V2, II, and the rhythm strip, within roughly three to four seconds, then lingered longer on those regions while virtually ignoring low-yield segments, such as the TP interval [4, 21, 22, 24, 27] (Table 2). Their scan paths were short and orderly, with the fewest overall fixations and revisits of any group [4, 21, 22, 33, 35]. Heat-map analyses have revealed a densely clustered gaze confined to key leads and a consistently systematic revisit pattern [4, 24, 27]. New data reinforce this pattern. In a pilot comparison, experts made fewer fixations (≈68 vs. 144) and completed reads faster (~ 108 s vs. ~ 205 s) than residents; they also showed smaller pupil dilation changes, consistent with lower cognitive load [36]. This focused search behavior allows experts to complete each tracing rapidly, underscoring the tight link between visual efficiency and high-level ECG expertise.

**Table 2:** Eye-tracking data of the included studies

First author, year	Fixation duration on each part of an ECG	Accuracy rate	Main results
Alahmadi et al. (2019) [19]	Rhythm strip 3.85 ± 5.21 s; single complex aligned 1.82 ± 2.21 s; unaligned 1.62 ± 2.75 s; format effects p < 0.05	Rhythm strip: 93.33% CR, 6.66% FA; Single complex (aligned): 100% CR; Single complex (unaligned): 90% CR, 10% FA	Laypeople detect QT prolongation; rhythm strip boosts detection; gaze on first 5 complexes; ~ 4 AOIs/trial. Minimal training enabled reliable self-monitoring of drug-induced QT prolongation
Augustyniak et al. (2003, 2006) [20, 22]	Experts vs students: idle 73 ± 55 vs 88 ± 105 ms; total 5.5 ± 1.5 vs 6.2 ± 1.7 s; foveation QRS 38% vs 26%, TP 14% vs 25%; attention density 21.0 vs 16.0	NA	Experts focus 50% longer on QRS, students on TP; experts had higher attention density and more purposive scanpaths; shorter total time
Augustyniak et al. (2005) [21]	Foveation time 31 ± 12% vs 17 ± 10%; foveation points 6.1 ± 1.7 vs 9.2 ± 3.9; scanpath length 34.7 ± 5.1° vs 28.5 ± 6.6°; duration 3.6 ± 1.3 vs 5.7 ± 1.5 s	NA	Experts: fewer foveations, shorter scanpaths, faster completion; higher scanpath similarity (37%) than students (17%), focused strategy with wider-spaced fixations and finished faster than students
Bond et al. (2012) [25]	Time by lead: rhythm 26% (162 s), V1 13% (85 s), V2 11% (71 s), aVR 1% (7 s). Precordial leads prioritized	NA	Clinical scientist (ECG expert): 39.6 ± 11.6 s per ECG (AMI 39.4; LVH 41.6; LBBB 37.6). Fixations: rhythm strip 26% vs aVR 1% (p < 0.01); precordials > limb leads (p = 0.002). Heatmaps confirm a rhythm-strip–first pattern
Bond et al. (2014) [26]	Longest fixation: V1 (4.29 s), V2 (3.83 s), rhythm strip (3.47 s); Shortest: V4 (1.19 s). First fixation: Lead I (5.7 ± 3.8 s), V6 last (19.17 ± 6.4 s). r between first fixation and total duration (r = 0.67)	Mean 63 ± 16%; κ = 0.56 (STEMI 79%, arrhythmias 71%)	21 experts showed moderate inter-rater agreement (Fleiss’ kappa = 0.56), highest for STEMI (79%) and arrhythmias (71%). Accuracy correlated with age (r = 0.67), experience (r = 0.59)
Bond et al. (2015) [23]	Heatmaps: focus on II, V2, V1	NA	Single expert (Childers): prioritized II, V2, V1 (expert focus) as shown by heatmap
Bortolotti et al. (2025) [36]	Experts fewer fixations 67.7 ± 25.7 vs 143.7 ± 29.9; faster interpretation time 107.6 ± 32.8 s vs 205.31 ± 57.43 s; smaller pupil-dilation change 4.8% ± 2.0 vs 10.5% ± 4.2 (all p < 0.001 except pupil p = 0.015)	PGY1 73.33% ± 4.34; PGY2 77.77% ± 3.85; PGY3 84.44% ± 6.16; Experts 93.33% ± 9.42 (p < 0.001)	Expertise tracks fewer fixations, shorter times, lower load, higher accuracy; effects stronger with difficulty
Breen et al. (2014) [26]	Rhythm strip most fixated (9.6 s); lead I least (0.1 s); Avg. interpretation: 307.9 s (range: 132.1–595.8 s)	Group A (eye-tracking feedback): 63 ± 12.38%; Group B (control): 67 ± 10.35% (p = 0.32)	Meantime 307.9 s (≈13.2–59.5 s/ECG); rhythm strip most fixated, lead I least. Duration correlated with performance (r = -0.81). eye-tracking-feedback: 75% improved (3–32%) vs 71% control (1–18%), ns (p = 0.32); ET rated 8/10 and perceived to boost competency
Broadbent et al. (2013) [27]	ECG 1 (AV block): Experts 0.62 s first fixation, 1.49 s total, 4.67 revisits vs. Novices 0.29 s, 0.65 s, 1.64 revisits ECG 2 (NSR): Experts 3.66 s to AOI, 1.20 s total vs. Novices 8.49 s, 0.53 s ECG 3 (anterior MI): Experts 9.13 s total, 30.57 revisits vs. Novices 4.63 s, 15 revisits ECG 4 (inferior MI): Experts 5.09 s, 17.33 revisits vs. Novices 3.90 s, ~ 12 revisits	NA	Experts reached diagnostic AOIs faster and revisited them more; novices relied on bottom-up salience. Heatmaps showed experts’ broader, targeted gaze, identifying diagnostic areas faster with more systematic patterns
Davies et al. (2016) [28]	Correct group: Shorter fixations, fewer on V2 (7.3 vs. 13.4 fixations) Incorrect group: Longest on rhythm strip II (3.68 s, 13.4 fixations) Layout A: Correct group longest on V2 (1.83 s), least on aVR (1.7 fixations) Layout B: Longest on V5 (correct: 2.28 s, incorrect: 3.55 s), least on aVR (correct: 1.7, incorrect: 2.3 fixations)	Overall: 9–73%. Professionals > students (p < 0.01). Hyperkalemia: 6.3%, VT: 84%	Correct group: shorter/fewer fixations (e.g., V2 7.3 vs 13.4 fixations); professionals > students (p < 0.01). Accuracy varied: VT 84%, hyperK 6.3%. Transition patterns differed in 5/11 ECGs
Davies et al. (2018) [29]	Correct group: Focused on V1, V2; Incorrect: More on aVL. Incorrect: 2307 transitions vs. Correct: 2146. Lead I fixation shorter for correct (p = 0.002)	STEMI: 53%, Atrial flutter: 84%, Hyperkalemia: 6%, VT: 84%, LBBB: 75%, NSR: 77%	Correct: focused V1/V2; incorrect: more aVL. Fewer transitions (2146 vs 2307). Shorter Lead I fixation (p = 0.002). Accuracy: STEMI 53%, aflutter 84%, hyperK 6%, VT 84%, LBBB 75%, NSR 77%
Davies et al. (2019) [30]	No difference in fixation duration/count with history. Grid-level transitions varied by history and accuracy	Overall: 64 ± 27%. Significant differences for LBBB, STEMI, AF (p < 0.05)	Clinical history shifted grid-level transitions but not overall accuracy (64 ± 27%). Significant accuracy differences for LBBB/STEMI/AF (p < 0.05). Fixation count/duration stable; systematic transitions aligned with accuracy
Scherff et al. (2024) [13]	NA	NA	9-min expert gaze video with commentary yielded small, non-significant learning gains (d = 1.60). Student interest and initial case performance predicted post-training scores. EYE-ECG rated engaging; pausing at critical points suggested for improvement
Sibbald et al. (2015) [31]	Checklist (CH): 88 ± 71 fixations, 32 ± 21 s verification, 13.1 ± 8.8 MP scanpath Analytic Prompt (AP): 52 ± 37 fixations, 21 ± 15 s, 7.9 ± 4.8 MP CH: Higher intra-lead saccades (p < 0.05)	CH: 0.27 ± 0.53 errors corrected/ECG AP: 0.04 ± 0.43 errors corrected/ECG (p = 0.01)	Checklist verification increased error correction (0.27 ± 0.53 vs 0.04 ± 0.43 per ECG; p = 0.01) with more analytical scanning (88 vs 52 fixations; 32 vs 21 s; higher intra-lead saccades)
Sqalli et al. (2022) [33]	Fixations count per participant (for all ECGs) [median + IQR]: Medical students: 2829 ± 1411/Technicians: 2535 ± 301/Nurses: 2444 ± 1031/Fellows: 2135 ± 579 Consultants: 138 ± 794 Fixation count per lead for each ECG: Medical students: 9.93 ± 5.01/Technicians: 10.83 ± 1.27/Nurses: 9.49 ± 3.96/Fellows: 9.12 ± 2.52/Consultants: 6.57 ± 3.95 ECG lead revisitation per participant [Mean (μ) SD (σ)]: Medical students: 3.61 ± 0.06/Technicians: 3.25 ± 1.60/Nurses: 2.90 ± 0.85/Fellows: 2.55 ± 0.67/Consultants: 2.01 ± 0.98	Consultants: 97.8%, Students: 52.2%, Fellows: 87%, Technicians: 70%, Nurses: 63%	Accuracy differed across groups (χ^2^, p = .02; Cramér’s V = 0.36, weak). Consultants had highest accuracy with fewest fixations; students had the most; nurses/technicians/fellows were intermediate. Total fixations differed (Kruskal–Wallis p = .03). Lead revisits were highest in technicians, lowest in consultants, with others between; η^2^ = 0.36 (moderate)
Sqalli et al. (2023) [32]	Total fixations: 45,917, clustered into 15,373 groups. Fixation count and duration strongly correlated (r = 0.92)	Four models were compared, with a baseline model achieving an average accuracy of 21% SVM: Accuracy ~ 65%; CNN: Accuracy: ~ 43%; Logistic Regression: Accuracy ~ 86%: Linear Regression: Accuracy ~ 92%	Linear regression predicted expertise best (accuracy: 92%). Fixation count, duration, revisitations highly correlated (r = 0.92). Outperformed SVM (~ 65%), CNN (~ 43%). Eye-tracking with ML effectively assessed visual expertise
Sqalli et al. (2021) [34]	**Top leads (fixation count & time):** Most: II—54 fixations; 2727 ms, then V5, V1, V3/ Least: V4—6 fixations; 312 ms, then V6, aVR.**Time to first fixation (TTFF):** Fastest: II—4225 ms; next aVL—3602 ms**/** Slowest: V4—19 460 ms; V6—18 641 ms **Revisitation frequency:** Highest: V2/V3/aVF ≈4 × **/** Lowest: V6/V4/aVR ≈1 × **Fixation count by rhythm type:** Rhythm strip vs short leads differ for NSR (p = 0.010) and AFL (p = 0.015). No differences for WPW, VT, LBBB, STEMI, AV block (all p > 0.1)	Overall: 55.63 ± 4.63%. NSR: 81%, LBBB: 31%	Students tended to start on II, V5, and V1, with gaze patterns varying by ECG. Fixation durations were left-skewed around ~ 100 ms, with bimodality in some (e.g., AFL). Fixation count correlated with duration (r = 0.81); TTFF correlated negatively with duration (r = − 0.36) and AOI revisits (r = − 0.44)
Wood et al. (2013) [4]	Time to abnormality: Experts 3.43 ± 2.01 s, Novices 6.62 ± 4.14 s Time to diagnosis: Experts 19.52 ± 8.48 s, Novices 37.29 ± 17.94 s Search rate: 2.40 ± 0.14 (both)	Mean accuracy from 8: Novices: 2.90 ± 1.68/ Experts: 6.05 ± 1.17	Experts diagnosed faster, more confidently, fixating critical leads twice as fast (3.43 vs. 6.62 s). Novices scanned inefficiently. Clinical history had no accuracy effect (experts: 6.05/8, novices: 2.90/8). No difference in search rate
Wu et al. (2021) [35]	Attendings: Focused, shorter times. Students: Scattered, slower. Residents: Structured but variable	From 10: Attendings: 8; Residents: 7; Students: 5	Interpretation time (H(2) = 16.637, p < 0.001) and accuracy (H(2) = 18.431, p < 0.001) differed. Experts used rapid, critical-area pattern recognition (8/10). Residents 7/10; students 5/10 -slower, more systematic, higher cognitive load

*AF* Atrial Fibrillation, *AOI* Area of Interest, *AV block* Atrioventricular Block, *ECG* Electrocardiogram, *JND* Just Noticeable Difference, *LBBB* Left Bundle Branch Block, *LVH* Left Ventricular Hypertrophy, *MI* Myocardial Infarction, *NSR* Normal Sinus Rhythm, *QT* QT Interval, *RBBB* Right Bundle Branch Block, *STEMI* ST-elevation myocardial infarction, *SVT* Supraventricular Tachycardia, *TP* TP Segment, *VF* Ventricular Fibrillation, *VT* Ventricular Tachycardia, *WPW* Wolff–Parkinson–White Syndrome

### Gaze behavior and visual efficiency among novices

Novice interpreters, including medical students, early residents, and laypersons, displayed an opposite pattern (Table 2). Their gaze was dispersed, producing nearly twice as many fixations and revisits as experts (about 2800 vs. 1400 per ten ECGs) [33]. The time to first fixation on abnormal leads often exceeded six seconds [4], and the overall interpretation times are substantially prolonged [4, 35]. Scan paths revealed frequent detours to visually prominent yet diagnostically marginal waveform segments, such as rhythm strips or central leads, especially in early scanning [26, 27, 35]. These patterns reflect a predominantly bottom-up, reactive search strategy common among novices [27–29] and underscore the need for structured training to promote more efficient, expert-like visual behavior. Moreover, compared to experts, residents required more fixations and longer viewing time to reach a decision [36], consistent with the dispersed novice-like pattern described above.

### Diagnostic accuracy

Twelve studies examined diagnostic accuracy through either the percentage of correct interpretations or inter-rater agreement statistics [4, 19, 24, 26, 28–35]. Across the included studies, diagnostic accuracy consistently varied by experience level, with experts outperforming the novices. Among experts, the highest reported accuracy was 97.8% [33], whereas novice medical students demonstrated accuracy rates as low as 9% in specific ECG categories [28]. For example, expert emergency physicians scored 6.05 out of 8, whereas medical students scored 2.90 out of 8, underscoring the performance gap [4]. In addition, accuracy followed a clear experience gradient: experts 93.3%, PGY-3 84.4%, PGY-2 77.8%, PGY-1 73.3% (p < 0.001) [36]. These findings reinforce the role of experience in achieving consistent and accurate ECG interpretation. The detailed accuracy metrics across the study and participant groups are presented in Table 2.

Across the five prespecified studies [4, 28, 33, 35, 36], the direction of effect favored experts in every case. Four studies reported groupwise accuracy that could be normalized: experts 93.3% vs PGY-1 73.3% (Δ = 20.0 pp); 6.05/8 vs 2.90/8 (76.0% vs 36.3%; Δ = 39.7 pp); consultants 97.8% vs students 52.2% (Δ = 45.6 pp); and attendings 8/10 vs students 5/10 (80% vs 50%; Δ = 30.0 pp). Davies et al. (2016) supported the same direction but lacked compatible denominators, so it contributes direction only. The median within-study difference across the four studies was ~ 34.9 percentage points (range 20.0–45.6), and excluding the largest and smallest Δ left the conclusion unchanged.

### Educational applications of eye-tracking in ECG interpretation

Several studies have incorporated interventions, such as visual training, structured checklists, or eye-tracking-based feedback. For example, Sibbald et al. found that checklists significantly improved error detection compared with open-ended prompts (0.27 vs. 0.04 errors per ECG, p = 0.01), supported by eye-tracking evidence of deeper visual scrutiny [31]. Scherff et al. implemented a video-based intervention in which students viewed expert gaze patterns synchronized with clinical reasoning audio [13]. While the post-test accuracy gains were not statistically significant, a large within-group effect size (*d* = 1.60) suggests educational benefits [13]. Overall, the effects were mixed: brief, single-session modeling/feedback produced small or non-significant gains (compared to checklist-based verification, which improved error detection and within-lead scrutiny).

Breen et al. also tested eye-tracking-based feedback in a randomized design but found no significant improvement over traditional teaching (p = 0.32), although interpretation duration was strongly correlated with diagnostic performance (r = −0.81) [26]. Although not an educational intervention, Sqalli et al. (2023) developed a machine learning model using eye-tracking features to predict ECG interpretation expertise with 94% accuracy, highlighting the potential of gaze data for assessment and personalized feedback design [32].

### Association between gaze metrics and diagnostic performance

At least eight studies have established quantitative links between gaze behavior and diagnostic accuracy [4, 24, 27, 28, 31, 34–36]. Key metrics such as fixation duration, dwell time, and scan-path efficiency were consistently correlated with performance. For example, Bond et al. reported r = 0.59 between experience and accuracy, and r = 0.67 between time to first fixation on a lead and the total fixation duration on that lead [24]. Wu et al. found that accuracy improved with longer dwell time on abnormal leads [35]. Wood et al. showed that experts fixated earlier on critical leads and diagnosed them more accurately [4]. Davies et al. and Broadbent et al. linked systematic visual behavior to higher accuracy [27, 28]. Sibbald et al. observed that checklists increase analytical scanning and reduce errors [31]. Sqalli et al. reported strong correlations between eye-tracking metrics and ECG interpretation behavior among medical students, including fixation count (r = 0.81), fixation revisitation (r = 0.78), and dwell time (r = 0.53) [34]. Bortolotti et al.’s study also linked behavior to performance: fewer fixations and shorter interpretation time co-occurred with higher accuracy across training levels, strengthening the view that efficient gaze supports correct decisions [36]. ‬‬‬‬‬‬‬‬‬‬‬

## Discussion

To our knowledge, this is the first ECG-specific systematic synthesis integrating lead-level gaze, diagnostic accuracy, and engineering reporting. Our ECG-specific focus extends prior general eye-tracking reviews by linking lead-level AOIs to performance, exposing device/detector heterogeneity, and summarizing within-study accuracy differences. Overall, experts exhibited shorter interpretation times, more targeted, longer fixations on diagnostically relevant leads, and markedly higher accuracy than novices. Across the studies with extractable groupwise data, experts outperformed less-experienced readers by a median of ~ 35 percentage points (range 20.0–45.6), aligning with more efficient gaze. Moreover, efficient gaze, earlier fixation on diagnostic leads, and fewer targeted fixations correlated with faster time-to-diagnosis. However, gaze is a process marker, not an outcome; so, our inferences are associational, not causal. Clinical value should be shown as durable gains in accuracy and time-to-diagnosis on authentic cases. Additionally, several studies explored the potential of gaze-informed educational interventions such as structured checklists and expert gaze modeling to enhance interpretive accuracy. Mixed educational results suggest the need for targeted, task-aligned interventions and prospective validation before their routine use.

Findings support the dual-process reasoning model, in which novices rely on analytical rule-based strategies, while experts increasingly use intuitive pattern-recognition approaches [35, 37]. However, it can also increase the susceptibility to error if it is not balanced by analytical reflection when cases are atypical or ambiguous. Eye-tracking heatmaps illustrate this distinction, with novices displaying more dispersed fixations [27], mirroring the patterns seen in radiology studies comparing expert and novice readers [38]. This distinction also reflects the concept of *unconscious competence*, wherein experts perform complex tasks with minimal deliberate effort owing to their extensive experience [39].

Students commonly begin with central leads and the rhythm strip (e.g., lead II), using them as anchors for systematic interpretation [34, 40]. However, their interpretation often shifts when waveform abnormalities attract attention, leading to increased revisits and deviation from systematic scanning, a reactive pattern that contrasts with the selective attention strategies used by experts [34]. Davies et al. described this as a “reactive” interpretation style, in which attention is driven by noticeable abnormalities [40]. Although experts may use this approach selectively, it is more commonly observed among novices, who are easily diverted by prominent waveform changes [34, 40]. In addition, while traditional ECG teaching emphasizes a step-by-step examination of waveform components for abnormalities, experts tend to rely on structured diagnostic reasoning schemes based on organized clinical knowledge [26, 41]. ‬‬‬‬‬‬‬‬‬‬‬‬‬‬‬‬‬‬‬‬‬‬‬‬‬‬‬‬‬‬‬‬‬‬‬‬‬‬‬‬‬‬‬‬‬‬‬‬‬‬‬‬‬‬‬‬‬‬‬‬‬‬‬‬‬‬‬‬‬‬‬‬‬‬‬‬‬‬‬‬‬‬‬‬‬‬‬‬‬‬‬‬‬‬‬‬‬‬‬‬‬‬‬‬‬‬‬‬‬‬‬‬‬‬‬‬‬‬‬‬‬‬‬‬‬‬‬‬‬‬‬‬‬‬‬‬‬‬

Eye-tracking metrics, such as fixation duration, fixation count, and time to first fixation, are common proxies for cognitive processing [42, 43], but their meanings are task-dependent. Longer fixations may signal expertise in fields such as chess and art. However, in low-demand tasks such as driving, they can reflect disengagement [28, 43]. This underscores the importance of interpreting the gaze data in the context of ECG expertise. In ECG interpretation, some conditions require attention to specific leads, whereas others, such as STEMI, require cross-referencing of multiple regions [28]. Therefore, interpreting gaze patterns meaningfully also depends on whether viewers engage with leads in a diagnostically informative manner. Wood et al. observed that experts were twice as fast as novices in fixating on critical leads and required significantly less time to reach a diagnosis once abnormalities were detected [4].

Several studies in this review identified variations in lead prioritization during ECG interpretation. For instance, Breen et al. observed that participants focused most heavily on the rhythm strip, whereas lead I received the least attention [26]. Likewise, Broadbent et al. found that expert nurses revisited critical leads like the anterior and inferior leads more frequently and for longer durations than novice nurses, highlighting a top-down knowledge-driven visual strategy [27]. Augustyniak et al. reported that experts fixated more intensely on QRS complexes and displayed higher attention density in diagnostically informative regions, whereas students spent excessive time in the TP segment and non-essential areas [20]. There are many different methods of teaching ECG interpretation that vary in approach and duration [44]. These methods also differ between countries and institutions, as well as the medical discipline to which the practitioner belongs [45].

Educational findings are mixed. Checklists used at the verification stage improved error detection (0.27 vs 0.04 errors per ECG, p = 0.01) and eye-tracking confirmed deeper within-lead scrutiny [31]. In contrast, single-session modeling with expert-gaze video or brief eye-tracking feedback produced small or non-significant accuracy gains [13, 26]. Recent data help explain what works: experts combined higher accuracy with fewer fixations and shorter interpretation times, alongside smaller pupil-dilation changes, and performance rose stepwise from PGY-1 to consultant [36], suggesting that repeated, task-aligned feedback targeting these gaze features is more likely to yield a durable benefit. Main barriers are cost, setup time, workflow fit, and data privacy; a practical path is to use shared screen-based trackers at 60 to 120 Hz with brief calibration, standard lead-area maps, and a simple I-VT detector during verification.

Our results point to a practical engineering path. Use a small set of gaze measures: how quickly the reader looks at key leads first, how long they look at each area, how often they return, and how direct the scan is. Tie these measures to fixed lead areas on the 12-lead display so training software can deliver clear, objective feedback, such as “You skipped V2 before deciding” or “Your scan was too long on non-critical leads” [34]. ‬‬‬‬‬‬‬‬‬‬‬‬‬‬‬‬

To make studies comparable across sites, report a short data-quality set including device model and type, sampling rate, accuracy, and calibration method, screen settings, how gaze timing is matched to what appears on screen, the detector used and its thresholds, data loss, and the exact lead-area templates, as recommended in eye-tracking methodology guidance [46, 47]. Use a few shared test cases with the same ECGs and the same lead maps. Then teams can test if simple user-interface prompts, such as a brief verification checklist at the end of reading, improve gaze patterns and accuracy rather than reflecting hardware or software differences [31].

Beyond descriptive group comparisons, recent engineering work has used the same eye-tracking features for automated expertise classification. Bokhari et al. applied neuromorphic architectures (SNN, SCNN, RSNN, SCLSTM) to ECG eye-movement features and achieved > 90% accuracy, with the best model reaching 95.6% [48]. Although clinical groups were not directly compared, these results show that gaze data can support machine-learning systems that recognize interpretive skill. Such approaches outline a path from descriptive metrics to scalable assessment tools; however, external validation and prospective studies remain necessary. Accordingly, we present gaze targets as candidate metrics for training/assessment, not validated performance standards.

Cross‐disciplinary work shows that eye-tracking (and related process-tracing) indexes attention, cognitive load, and integration in language processing [49], differentiates expert–novice pathways in clinical reasoning [50], and, in multimedia learning, leverages signaling/segmentation to externalize expert attention [51]. Tien et al. reviewed 24 studies across surgical and clinical tasks (laparoscopy, radiology, pathology, anesthesia, nursing) and showed that eye-tracking reliably separates experts from novices using fixation count, dwell time, and gaze distribution [18]. They also highlighted dual value for assessment and training, including gaze-based feedback to accelerate procedural learning [18]. These findings support eye-tracking as both a diagnostic and educational tool for ECG.

This study had several limitations. First, substantial heterogeneity in study design, participant experience, and technical specifications limited direct comparisons and favored a narrative over a quantitative synthesis. Devices and sampling rates varied widely (60–1000 Hz), and calibration accuracy, detector choice/thresholds, and screen/gaze-timing alignment were inconsistently reported. Second, many studies enrolled small samples (including n = 1), and three were judged high risk of bias. Third, the variation in ECG tasks, from rhythm-strip identification to full 12-lead analysis, means that gaze behavior may be task-dependent, and may not uniformly translate to real-world clinical settings. We did not evaluate cost-effectiveness or implementation barriers (hardware/maintenance costs, setup time, workflow fit, faculty time, data governance), which may constrain uptake and should be assessed alongside accuracy and time-to-diagnosis. Fourth, most evidence is observational or exploratory, with few randomized trials and limited data on durable educational benefit. Finally, publication bias cannot be ruled out, as positive or innovative results are more likely to be published. Given small samples, predominantly observational designs, and heterogeneous hardware and analysis parameters, these findings should be viewed as hypothesis-generating; prospective, adequately powered studies are needed before claiming reliability at scale.

### Future directions

To enable cumulative evidence, future work should adopt common design and reporting standards, including clearly reporting tracker characteristics and calibration procedures, predefining diagnostic AOIs, controlling case order and presentation, and specifying task constraints. Studies should be adequately powered and, where feasible, multi-site and longitudinal to test the durability of effects. A harmonized set of gaze metrics, including TTFF to diagnostic AOIs, fixation count, and dwell proportion on AOIs, revisitations, scan-path efficiency, and time-to-diagnosis, would facilitate comparison across studies. An ECG-specific core outcome set should prioritize diagnostic accuracy and time-to-diagnosis, with cognitive load, confidence, and retention as secondary outcomes. Educational trials should compare gaze-guided verification and brief expert-modeling against active, time-matched controls. Preregistration, open stimuli/AOI definitions, and shared analysis code are recommended to improve transparency and reproducibility.

## Conclusion

Expert ECG readers consistently show focused, efficient gaze patterns that align with faster, more accurate interpretation. Eye-tracking metrics, such as fixation behavior and scan-path efficiency, are promising indicators for assessment and for designing feedback-based training. Because current evidence is largely associational across heterogeneous methods, adoption should await prospective studies demonstrating durable gains in accuracy and time-to-diagnosis and feasibility in real settings. Standardized reporting and multicenter trials will be key to realizing eye-tracking’s value in ECG education and evaluation.

## Supplementary Information

Supplementary material 1. Table S1. Search strategy. Table S2. Technical eye-tracking reporting across studies.

## Data Availability

Availability of data and materials: Data sharing does not apply to this article, as no datasets were generated or analyzed during the current study.

## Abbreviations

AF: Atrial fibrillation
AOI: Area of interest
AV block: Atrioventricular block
CH: Checklist
CRR: Clinical reasoning report
ECG: Electrocardiogram
FY2: Foundation Year 2
JND: Just noticeable difference
LBBB: Left bundle branch block
LVH: Left ventricular hypertrophy
MI: Myocardial infarction
NA: Not available
NSR: Normal sinus rhythm
QT: QT interval
RBBB: Right Bundle Branch Block
ROB: Risk of bias
STEMI: ST-elevation myocardial infarction
SVT: Supraventricular tachycardia
TAU: Treatment as usual
TP: TP segment
VF: Ventricular fibrillation
VT: Ventricular tachycardia
WPW: Wolff–Parkinson–White syndrome
