# Supplementary material for: A systematic review of eye-tracking technology in electrocardiogram interpretation research
Source: Eur J Med Res. 2025 Dec 5;31:50. doi: 10.1186/s40001-025-03635-8 (PMC12797453; doi:10.1186/s40001-025-03635-8)
Supplement: Supplementary file 1 — Supplementary material 1. Table S1. Search strategy. Table S2. Technical eye-tracking reporting across studies. [file 40001_2025_3635_MOESM1_ESM.docx]

Table S1. Search strategy

| **Databases (Field)** | **Search query** |
| --- | --- |
| **Medline (Title/Abstract)** | ("Eye-Tracking Technology" OR "Eye Movement Measurements" OR "Eye Movement*" OR "Eye-Movement" OR "Eye Tracking" OR "Eye-Tracking" OR "Eyetracking" OR "Gaze-Tracking" OR "eye track*" OR "eyetrack*" OR "Gaze Tracking" OR "GazeTracking" OR "Visual Search" OR "Eye Fixation" OR "Ophthalmic Fixation" OR "Functional Vision Testing" OR "Visual System" OR "Visual Tracking" OR "visual behavior" OR "visual behaviour" OR "Visual Display*" OR "Visual Stimulation" OR "visual interpretation" OR "Smooth Pursuit Eye" OR "Orienting Reflex" OR "oculomotor" OR "fixation tracking" OR "saccade*" OR "Saccadic" OR "eye task" OR "visual track" OR "visual process*" OR "Ocular Focusing" OR "Ocular Fixation" OR "eye gaze" OR "Eye Movement Measurements" OR "eye gaz*" OR "fixation*" OR "saccade*" OR "looking behaviour" OR "looking behavior" OR "gaze follow*" OR "gaze behavior" OR "gaze behaviour") AND (“Electrocardiogram*” OR “ECG*” OR “EKG*” OR Cardiography OR Cardiographies OR Electrocardiograms OR Electrocardiograph OR Electrocardiographs OR "Electrocardiogr*" OR "Elektrocardiogr*" OR "Electro cardiogr*" OR "Elektro cardiogr*" OR "Electrocardiography") |
| **EMBASE (Title, Abstract or Author keywords)** | ('Eye Movement*' OR 'Eye-Movement' OR 'Eye Tracking' OR 'Eye-Tracking' OR 'Eyetracking' OR 'Gaze-Tracking' OR 'eye track*' OR 'eyetrack*' OR 'Gaze Tracking' OR 'GazeTracking' OR 'Visual Search' OR 'Eye Fixation' OR 'Ophthalmic Fixation' OR 'Functional Vision Testing' OR 'Visual System' OR 'Visual Tracking' OR 'visual behavior' OR 'visual behaviour' OR 'Visual Display*' OR 'Visual Stimulation' OR 'visual interpretation' OR 'Smooth Pursuit Eye' OR 'Orienting Reflex' OR 'oculomotor' OR 'fixation tracking' OR 'saccade' OR 'Saccadic' OR 'eye task' OR 'visual track' OR 'visual process*' OR 'Ocular Focusing' OR 'Ocular Fixation' OR 'eye gaze' OR 'Eye Movement Measurements' OR 'eye gaz*' OR 'fixation*' OR 'saccade*' OR 'looking behaviour' OR 'looking behavior' OR 'gaze follow*' OR 'gaze behavior' OR 'gaze behaviour') AND ('Electrocardiogram*' OR 'ECG*' OR 'EKG*' OR Cardiography OR Cardiographies OR Electrocardiograms OR Electrocardiograph OR Electrocardiographs OR 'Electrocardiogr*' OR 'Elektrocardiogr*' OR 'Electro cardiogr*' OR 'Elektro cardiogr*' OR 'Electrocardiography') |
| **Web of Science (Topic)** | ("Eye-Tracking Technology" OR "Eye Movement Measurements" OR "Eye Movement*" OR "Eye-Movement" OR "Eye Tracking" OR "Eye-Tracking" OR "Eyetracking" OR "Gaze-Tracking" OR "eye track*" OR "eyetrack*" OR "Gaze Tracking" OR "GazeTracking" OR "Visual Search" OR "Eye Fixation" OR "Ophthalmic Fixation" OR "Functional Vision Testing" OR "Visual System" OR "Visual Tracking" OR "visual behavior" OR "visual behaviour" OR "Visual Display*" OR "Visual Stimulation" OR "visual interpretation" OR "Smooth Pursuit Eye" OR "Orienting Reflex" OR "oculomotor" OR "fixation tracking" OR "saccade*" OR "Saccadic" OR "eye task" OR "visual track" OR "visual process*" OR "Ocular Focusing" OR "Ocular Fixation" OR "eye gaze" OR "Eye Movement Measurements" OR "eye gaz*" OR "fixation*" OR "saccade*" OR "looking behaviour" OR "looking behavior" OR "gaze follow*" OR "gaze behavior" OR "gaze behaviour") AND ("Electrocardiogram*" OR "ECG*" OR "EKG*" OR Cardiography OR Cardiographies OR Electrocardiograms OR Electrocardiograph OR Electrocardiographs OR "Electrocardiogr*" OR "Elektrocardiogr*" OR "Electro cardiogr*" OR "Elektro cardiogr*" OR "Electrocardiography") |
| **Scopus (Title)** | TITLE ( ( "Eye-Tracking Technology" OR "Eye Movement Measurements" OR "Eye Movement*" OR "Eye-Movement" OR "Eye Tracking" OR "Eye-Tracking" OR "Eyetracking" OR "Gaze-Tracking" OR "eye track*" OR "eyetrack*" OR "Gaze Tracking" OR "GazeTracking" OR "Visual Search" OR "Eye Fixation" OR "Ophthalmic Fixation" OR "Functional Vision Testing" OR "Visual System" OR "Visual Tracking" OR "visual behavior" OR "visual behaviour" OR "Visual Display*" OR "Visual Stimulation" OR "visual interpretation" OR "Smooth Pursuit Eye" OR "Orienting Reflex" OR "oculomotor" OR "fixation tracking" OR "saccade*" OR "Saccadic" OR "eye task" OR "visual track" OR "visual process*" OR "Ocular Focusing" OR "Ocular Fixation" OR "eye gaze" OR "Eye Movement Measurements" OR "eye gaz*" OR "fixation*" OR "saccade*" OR "looking behaviour" OR "looking behavior" OR "gaze follow*" OR "gaze behavior" OR "gaze behaviour" ) AND ( "Electrocardiogram*" OR "ECG*" OR "EKG*" OR cardiography OR cardiographies OR electrocardiograms OR electrocardiograph OR electrocardiographs OR "Electrocardiogr*" OR "Elektrocardiogr*" OR "Electro cardiogr*" OR "Elektro cardiogr*" OR "Electrocardiography" ) ) |
| **Eric** | Various permutations of keywords: "Eye Movement", "Eye-Movement", "Eye Tracking", "Eye-Tracking", "Gaze-Tracking", "Gaze Tracking", "Eye Fixation", "Ophthalmic Fixation", "Visual Tracking", "Visual Stimulation", "visual interpretation", "Orienting Reflex", "oculomotor", "fixation tracking", "Saccadic", "eye task", "visual track", "Ocular Focusing", "Ocular Fixation", "eye gaze", "saccade*", "Electrocardiogram", "ECG", "EKG", "Cardiography", "Cardiographies" Electrocardiogram, Electrocardiograph, "Electrocardiography") |
| **IEEE Xplore (Abstract)** | ("Abstract":"Eye Tracking" OR "Abstract":Eyetracking OR "Abstract":"Eye Movement*" OR "Abstract":gaze OR "Abstract":fixation* OR "Abstract":saccade*) AND ("Abstract":Electrocardiogram* OR "Abstract":ECG* OR "Abstract":EKG* OR "Abstract":Electrocardiograph* OR "Abstract":Electrocardiography) |
| **Pubpsych** | ("eye tracking" OR eyetrack* OR "eye movement*" OR gaze OR fixation* OR saccade*) AND (electrocardiogram* OR ECG* OR EKG* OR electrocardiograph* OR electrocardiography) |
| **Manual search in Google and Google Scholar** | Various permutations of keywords: "Eye Movement", "Eye-Movement", "Eye Tracking", "Eye-Tracking", "Gaze-Tracking", "Gaze Tracking", "Eye Fixation", "Ophthalmic Fixation", "Visual Tracking", "Visual Stimulation", "visual interpretation", "Orienting Reflex", "oculomotor", "fixation tracking", "Saccadic", "eye task", "visual track", "Ocular Focusing", "Ocular Fixation", "eye gaze", "saccade*", "Electrocardiogram", "ECG", "EKG", "Cardiography", "Cardiographies" Electrocardiogram, Electrocardiograph, "Electrocardiography") |

**Table S2.** Technical eye-tracking reporting across studies

| **Study (first author; year)** | **Device model** | **Device type** | **Sampling rate (Hz)** | **Calibration / accuracy** | **Fixation / saccade detector** |
| --- | --- | --- | --- | --- | --- |
| **Alahmadi; 2019** | Tobii X2–60 eye tracker and Tobii Studio 3.2 software | Screen-based (remote) | 60 | NR | Tobii Studio 3.2 used; detector NR |
| **Augustyniak; 2003, 2006** | Infrared reflection-based eye tracker  (OBER-2) | Head-mounted goggles | 750 | Total IR power 5 mW/cm²; **0.02°** angular resolution; notes on keeping goggle position fixed from calibration to measurement | Custom heuristic pre-processing; fixation detector not specified (NR) |
| **Augustyniak; 2005** | Infrared reflection-based eye tracker  (OBER-2) | Head-mounted goggles | 750 | Calibration rectangle noted; ~0.02° angular resolution | Custom processing (MATLAB); detector NR |
| **Bond; 2012** | Tobii X60 | Screen-based (remote) | 60 | Calibrated; accuracy NR | Tobii Studio (built-in); detector NR |
| **Bond; 2014** | Tobii X60 | Screen-based (remote) | 60 | Calibrated; accuracy NR | “Predefined computerized classification” (Tobii Studio) |
| **Bond; 2015** | Tobii X60 | Screen-based (remote) | 60 | NR | Tobii Studio (built-in); detector NR |
| **Bortolotti; 2025** | Tobii Pro Glasses 2 and Tobii Pro Lab software | Head-mounted | NR | NR | NR |
| **Breen; 2014** | Tobii X60 and the Tobii Studio 3 software | Screen-based (remote) | 60 | Calibration NR | Detector NR (Tobii Studio used) |
| **Broadbent; 2013** | Tobii T120 and Tobii Studio software | Screen-based (remote) | NR | Calibration NR | Detector NR (Tobii Studio mentioned) |
| **Davies; 2016** | Tobii 1750; Tobii X2-60 | Screen-based (remote) | NR (X2-60 nominal 60) | Pre/post calibration checks; <70% quality excluded | Tobii I-VT (default); min fixation 60 ms |
| **Davies; 2018** | Tobii 1750; Tobii X2-60 | Screen-based (remote) | NR | Calibration/accuracy NR | Detector NR |
| **Davies; 2019** | Tobii X2-60 and Tobii studio software version 3.2.0 | Screen-based (remote) | NR | Calibration/accuracy NR | Detector NR |
| **Scherff; 2024** | NR | NR | NR | NR | NR |
| **Sibbald; 2015** | BeGaze 2.4 (SensoMotoric Instruments GmbH,  Teltow) | Screen-based | NR | NR | NR |
| **Sqalli; 2022** | Tobii Pro X2-60 and iMotions version 8.1 software | Screen-based (remote) | 60 | Calibration performed; method NR | Detector NR |
| **Sqalli; 2023** | Tobii Pro X2-60 and iMotions version 8.1 software | Screen-based (remote) | 60 | NR | NR |
| **Sqalli; 2021** | Tobii Pro X2-60 and iMotions version 8.1  software | Screen-based (remote) | 60 | Calibration performed; method NR | Detector NR |
| **Wood; 2013** | ASL Mobile Eye | Wearable/glasses | 25 | NR | NR |
| **Wu; 2021** | Tobii Pro X3-120 | Screen-based (remote) | 120 | “Screen-based eye-tracker was calibrated”; method NR | NR |
